# The association between the degree of social vulnerability and maternal anemia and other adverse perinatal outcomes

**DOI:** 10.1002/pmf2.70250

**Published:** 2026-02-10

**Authors:** Rubymel J. Knupp, Christina T. Blanchard, Ariann Nassel, Ashley Shea, Lindsay S. Robbins, Jeff M. Szychowski, Ashley N. Battarbee, Akila Subramaniam

**Affiliations:** ^1^ Division of Maternal Fetal Medicine Department of Obstetrics and Gynecology University of Alabama at Birmingham Birmingham Alabama USA; ^2^ Center for Women's Reproductive Health University of Alabama at Birmingham Birmingham Alabama USA; ^3^ Regional Obstetric Consultants, Pediatrix Medical Group Jacksonville Florida USA; ^4^ Department of Biostatistics School of Public Health University of Alabama at Birmingham Birmingham Alabama USA; ^5^ Department of Health Policy and Organization School of Public Health, University of Alabama at Birmingham Birmingham Alabama USA; ^6^ Department of Obstetrics & Gynecology and Center for Maternal and Child Health Equity and Advocacy Eastern Virginia Medical School Norfolk Virginia USA

**Keywords:** anemia, pregnancy, social determinants, social vulnerability

## Abstract

**Objective:**

Maternal anemia in pregnancy is associated with many adverse maternal, fetal, and neonatal outcomes. The role of social determinants of health (SDOH) in the development of maternal anemia and other adverse outcomes is continually evolving. One such newer tool, the Social Vulnerability Index (SVI), measures an individual's community‐level SDOH, with higher SVI denoting higher vulnerability. Our objective was to evaluate the association between SVI, maternal anemia, and other adverse perinatal outcomes.

**Methods:**

Retrospective cohort study of pregnant individuals delivering singletons at a gestational age ≥23 weeks at a single center from 2014 to 2018. Patients with sickle cell disease, on anticoagulation, or with a PO Box, missing address, or residential address that could not be geocoded, were excluded. SVI scores were assigned to each patient based on their geographic home address and categorized into SVI tertiles: low (SVI 0–0.33), moderate (0.33–0.66), and high vulnerability (0.67–1.00). The primary outcome was anemia per ACOG guidelines (hemoglobin or hematocrit less than 11 g/dL and 33% in the first trimester, respectively, 10.5 g/dL and 32% in the second trimester, respectively, and 11 g/dL and 33% in the third trimester, respectively). Secondary outcomes included select labor outcomes, a composite of postpartum maternal morbidity, and a composite of neonatal morbidity. Multivariable logistic regression models estimated the association between moderate and high SVI and outcomes, compared to low SVI. The mean overall SVI score and SVI theme scores were compared for patients with and without anemia; individual SVI theme components were also analyzed. A mediation analysis using bootstrapping evaluated the contribution of anemia to the other adverse perinatal outcomes associated with SVI.

**Results:**

Of 13,951 individuals, 3989 (29%) lived in a low SVI area, 3976 (29%) lived in a moderate SVI area, and 5986 (43%) lived in a high SVI area. Patient characteristics varied significantly based on the SVI, as high SVI areas had patients who were younger, non‐Latinx Black, single, publicly insured, and more likely to have greater body mass index (BMI), chronic hypertension, and substance use issues; moderate SVI areas had more Latinx patients with diabetes; and low SVI areas had patients who were older, nulliparous, married, and privately insured. Compared to patients with low SVI, those with greater vulnerability had significantly higher odds of anemia (moderate SVI: aOR, 1.11 [1.00–1.24]; high SVI: aOR, 1.18 [1.05–1.32]). Preeclampsia, composite postpartum maternal morbidity, and neonatal morbidity also increased with increasing vulnerability. Patients with anemia had higher mean SVI scores overall and across the four SVI themes. In mediation analysis, 36% of the association between SVI and maternal morbidity was significantly mediated by anemia (*p* < 0.001), while 19% of the association between SVI and neonatal morbidity was mediated by anemia (*p* = 0.009).

**Conclusion:**

Patients living in moderate and high areas of social vulnerability are more likely to have anemia and other adverse perinatal outcomes. While some increase in the perinatal morbidity associated with increasing SVI is mediated through anemia, other factors and social determinants of health are likely also contributory.

## 1 INTRODUCTION

Anemia is the most common hematologic abnormality in pregnancy, affecting 40% of all pregnant persons worldwide and 48.8% by the third trimester alone [1, 2]. It is linked to significant maternal, fetal, and neonatal morbidity, including postpartum hemorrhage, maternal blood transfusion, preterm birth, cesarean delivery, longer hospital stays, and in offspring, perinatal death, low neonatal Appearance, Pulse, Grimace, Activity, Respiration (APGARs), and low birth weight [2, 3, 4, 5]. According to American College of Obstetricians and Gynecologists (ACOG), anemia in pregnancy is defined by trimester as hemoglobin or hematocrit less than 11 g/dL and 33% in the first trimester, 10.5 g/dL and 32% in the second trimester, and 11 g/dL and 33% in the third trimester, respectively [6, 7, 8].

In the United States, underrepresented communities are at increased risk of antenatal anemia, with Black persons having a two‐ to threefold greater rate of anemia than White persons [1, 8]. Social disparities may underly this difference in anemia rates among Black and White populations. Several risk factors such as poor iron diet, heavy menses, short‐interval pregnancy, diseases of malabsorption, and social determinants of health (SDOH; e.g., housing and lower socioeconomic class) contribute to anemia and other adverse perinatal outcomes [9, 10, 11]. In the non‐pregnant population, socioeconomic status is inversely related to anemia prevalence; while the general association with SDOH is recognized, the mechanisms and complex interplay of multiple SDOH factors remain areas of active and evolving investigation [12, 13, 14]. A measure for community‐level SDOH, the Social Vulnerability Index (SVI), is a publicly available integrated tool designed to assess a community's resources and preparedness to respond to external stressors and insults [15, 16, 17]. The SVI, a community‐level SDOH measure, is defined as the degree to which a community exhibits certain social conditions including high poverty, low percentage vehicle access, or crowded households that may affect the community's ability to prevent human suffering and financial loss in the event of a disaster [15, 16]. While high SVI has been associated with adverse pregnancy outcomes including peripartum cardiomyopathy, preterm birth, and diabetes, the impact of community‐level SDOH on anemia in pregnancy is not clear [13, 18, 19, 20, 21, 22, 23]. Thus, the aim of our study was to evaluate the association between community‐level social vulnerability, defined by the SVI, with maternal anemia and other adverse outcomes. Secondarily, we aimed to evaluate the contribution of maternal anemia to adverse pregnancy outcomes based on level of community‐level social vulnerability.

## 2 MATERIALS AND METHODS

After Institutional Review Board approval (IRB‐300002492), we performed a retrospective cohort study using an obstetric database of all individuals who delivered at a single center from 2014 to 2018. This large, validated database included deliveries over a 5‐year period—using a combination of validated data extraction from the electronic medical record as well as individual chart review by trained abstractors [24, 25]. Data were gathered through a manual chart review and validated coded data extraction [24, 25]. The data collection was performed by a research team consisting of maternal‐fetal medicine faculty and fellows, obstetric and gynecology residents, and fourth‐year medical students, all of whom were trained by the authors using prespecified rules [24, 25]. The abstraction process was validated by reviewing initial data subsets to ensure completeness and accuracy [24, 25].

**FIGURE 1:**
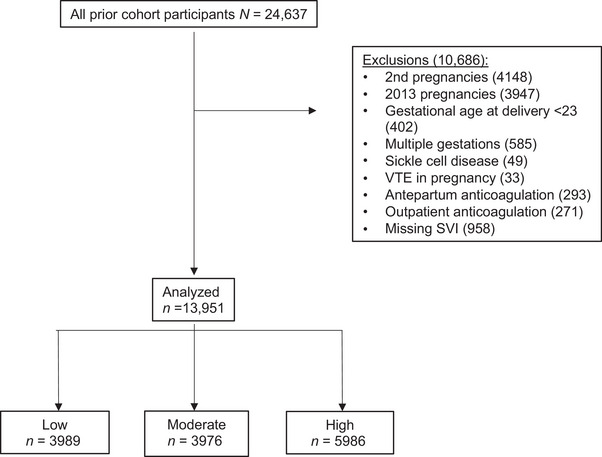
Flowchart of eligibility determination for inclusion in this secondary analysis.

For this analysis, we included patients with a singleton pregnancy who delivered at or above 23 weeks gestation between 2014 and 2018. Patients were excluded if they had sickle cell disease, were on any antepartum/outpatient anticoagulation, or reported a post office box as their primary place of residence, had a missing address, or an address that could not be geocoded (Figure 1). Sickle cell and anticoagulation were specifically excluded given their association with both anemia and adverse outcomes; other causes of anemia (i.e., megaloblastic anemia or thalassemia) are rare in our population and not available in our utilized database. Of note, only one pregnancy (the first pregnancy in the study period) per participant was used for analysis (Figure 1).

The primary exposure was a patient's neighborhood as represented by the SVI. The SVI, developed by the Centers for Disease Control and Prevention, is a well‐described tool that measures and maps the social vulnerabilities of communities, typically to help public health officials and urban planners prepare for and respond to emergencies [15, 16, 17, 21, 22, 23, 26]. An overall SVI score is calculated from a combination of 15 social factors obtained from census data, which are divided into four themes [15, 16]. Each theme receives a separate ranking as well as an overall ranking that includes all themes. Themes include socioeconomic status (below poverty level, unemployment, per capita income, no high school diploma), household characteristics (household members aged 65 or older or 17 or younger, household members with disabilities, and single‐parent households), racial and ethnic minority status, and housing type and transportation (multiunit structures, mobile homes, crowding, households with no vehicles) [15, 16]. The overall SVI is a percentile ranking scale of 0 to 1, with 0 being the least vulnerable and 1 the most vulnerable for a defined census‐tract geographical level (or small, relatively permanent statistical subdivisions of a county) [15, 16]. For our study, patient addresses at delivery were extracted from the electronic medical record. Addresses were geocoded using Esri's ArcPro 3.1, a Geographic Information System. The federal information processing standards (FIPS) code for each census tract associated with the geocoded address was then identified and used to link to the census tracts corresponding SVI score. We then classified these patients into tertiles based on the ranking scale: low (0 to 0.333), moderate (0.334 to 0.666), and high (0.667 to 1) from the least vulnerable to the most vulnerable. Utilizing tertiles as well as quartiles has been previously and widely reported [20, 27, 28, 29, 30]. For our secondary analysis, SVI scores—both composite overall and for each theme—were analyzed as a continuous variable rather than in tertiles.

The primary outcome was anemia defined as per ACOG guidelines: hemoglobin <10.5 g/dL or Hct < 32% on admission closest to delivery in the second trimester and hemoglobin <11 g/dL or Hct < 33% on admission, closest to delivery in the third trimester. Secondary outcomes included mode of delivery, delivery gestational age (GA), a postpartum maternal morbidity composite, and a neonatal morbidity composite. The postpartum maternal morbidity composite included one or more of the following: clinical diagnosis of endometritis, transfusion of packed red blood cells, unplanned operative procedures (such as wound exploration/drainage, exploratory laparotomy, hysterectomy, dilation and curettage, re‐operation on perineal lacerations, or any other procedure related to obstetric complications), wound/postoperative complications (such as superficial skin infection, cellulitis, fasciitis, deep wound infection, intraabdominal infection, and non‐infectious causes like hematoma, seroma, and dehiscence), postpartum readmission, postpartum intensive care unit (ICU) admission, and maternal death. The neonatal morbidity composite included one or more of the following: appearance, pulse, grimace, activity, respiration (APGAR) at 5 min < 4, neonatal intensive care unit (NICU) admission, and neonatal death. Details on definitions of these outcomes and sources of these data have been previously reported [24, 25].

Baseline maternal characteristics (i.e., age, BMI, race/ethnicity, insurance type, comorbidities) were compared across all three SVI ranking groups using Chi‐squared tests for categorical variables and one‐way ANOVA for continuous variables. Primary and secondary outcomes were then compared across the three social vulnerability ranking groups in a similar fashion. Multivariable logistic regression estimated the association between moderate and high SVI compared to low SVI. Initial multivariable models included maternal age, BMI, race, marital status, insurance, nulliparity, chronic hypertension, pregestational diabetes, gestational diabetes mellitus (GDM), tobacco use, illicit drug use, and aspirin use. Backward selection was used to generate parsimonious models including only those factors that remained statistically significant, and adjusted odds ratios (aORs) and 95% confidence intervals (CIs) were reported.

In a secondary analysis, we compared mean SVI scores (with 95% CIs) using Student's *t*‐test both for overall and for each theme between patients with and without anemia. Each SVI theme was further divided into individual components, which were compared between groups. For both overall composite SVI and SVI theme scores, adjusted ORs quantified the increased odds (crude and adjusted) of anemia for every 1 unit change in an SVI score to compare the relative contributions of each theme to anemia. Every 1 unit change referred to 0.1 of the original SVI scale and was rescaled by multiplying by 10 in order to aid in interpretation of the results. Finally, in order to evaluate the contribution of anemia to poor perinatal outcomes associated with SVI, we utilized a test for mediation using bootstrapping. Results were considered statistically significant at *p* < 0.05. All analyses were done with SAS 9.4.

## 3 RESULTS

Of 24,637 patients in the validated obstetric database, 13,951 pregnant patients met criteria for this analysis. Of these patients, 3989 (29%) had low, 3976 (29%) moderate, and 5986 (43%) high social vulnerability (Figure 1). Maternal characteristics by SVI group are presented in Table 1. Compared to low and moderate SVI groups, patients living in areas with a high SVI had the highest crude rate of younger, parous, non‐Latinx, Black, single, publicly insured, and the highest crude rate of greater BMI, chronic hypertension, drug and tobacco use (*p* < 0.001). Patients living in areas with moderate SVI had the highest crude rate of Latinx descent and diagnosis of pregestational or GDM (*p* < 0.001). Areas with the lowest SVI had the highest crude rate of older, nulliparous, married, and privately insured patients (*p* < 0.001).

**TABLE 1:** Maternal baseline characteristics/demographics.

	Low SVI (*n* = 3989) *N* (%)	Moderate SVI (*n* = 3976) *N* (%)	High SVI (*n* = 5986) *N* (%)	*p* value
Maternal age (years)	29.3 ± 5.7	27.1 ± 6.1	26.3 ± 6.0	<0.001
BMI prepregnancy (kg/m^2^)	28.0 ± 7.0	29.8 ± 8.2	30.1 ± 8.3	<0.001
Race/ethnicity				<0.001
Black	771 (19.3)	1643 (41.3)	3975 (66.4)	
White	2506 (62.8)	1440 (36.2)	887 (14.8)	
Hispanic	426 (10.7)	738 (18.6)	979 (16.4)	
Other	286 (7.2)	155 (3.9)	145 (2.4)	
Marital status				<0.001
Married	2454 (61.5)	1435 (36.1)	1178 (19.7)	
Single	1409 (35.3)	2400 (60.4)	4646 (77.6)	
Other	126 (3.2)	141 (3.6)	162 (2.7)	
Insurance status				<0.001
Private	2540 (63.7)	1221 (30.7)	913 (15.3)	
Public	1396 (35.0)	2709 (68.1)	5008 (83.7)	
Uninsured/self‐pay	53 (1.3)	46 (1.2)	65 (1.1)	
Nulliparous	1881 (47.2)	1749 (44.0)	2273 (38.0)	<0.001
Chronic hypertension	231 (5.8)	318 (8.0)	488 (8.2)	<0.001
Diabetes mellitus				
Pregestational	105 (2.6)	144 (3.6)	193 (3.2)	0.04
Gestational	305 (7.7)	356 (9.0)	441 (7.4)	0.01
Tobacco use in pregnancy	360 (9.0)	549 (13.8)	967 (16.2)	<0.001
Drug use in pregnancy	122 (3.1)	218 (5.5)	505 (8.4)	<0.001
Aspirin use in pregnancy	377 (9.5)	478 (12.0)	791 (13.2)	<0.001

*Note*: Data presented as mean ± standard deviation or *n* (%). Chi‐square and one‐way ANOVA tests were used for comparisons.

Abbreviation: SVI, Social Vulnerability Index.

Overall, 4080 (29.2%) in our cohort had anemia, details of which are presented in Table 2. A total of 2087 (34.9%) patients living in high SVI areas had anemia, 3976 (28.5%) living in moderate SVI areas had anemia, and 862 (21.6%) living in low SVI areas had anemia (*p* < 0.001). In crude analysis, patients living in areas with moderate SVI and high SVI had significantly higher odds of anemia as compared to those living in communities with low SVI (moderate SVI: OR, 1.44 [1.30–1.60]; high SVI: OR, 1.94 [1.77–2.13]). This relationship persisted after multivariable adjustments (moderate SVI: aOR, 1.11 [1.00–1.24]; high SVI: aOR, 1.18 [1.05–1.32]; Table 3). In fact, when evaluating SVI as a continuous variable, for each 1 unit change in SVI, the odds of anemia increased (aOR, 1.26; 95% CI, 1.09–1.45) (Table 5).

**TABLE 2:** Primary and secondary outcomes among SVI groups.

	SVI (low) (*n* = 3989) *N* (%)	SVI (moderate) (*n* = 3976) *N* (%)	SVI (high) (*n* = 5986) *N* (%)	*p* value
Primary outcome				
Anemia^a^	862 (21.6%)	1131 (28.5%)	2087 (34.9%)	<0.001
Secondary outcomes				
*Labor outcomes*				
Mode of delivery				<0.001
Vaginal	2632 (66.0%)	2677 (67.3%)	4177 (69.8%)	
Cesarean	1357 (34.0%)	1299 (32.7%)	1809 (30.2%)	
Delivery GA	38.3 ± 2.8	37.9 ± 3.4	37.9 ± 3.3	<0.001
*Postpartum maternal morbidity composite*	239 (6.0%)	336 (8.5%)	514 (8.6%)	<0.001
Endometritis	26 (0.7%)	25 (0.6%)	36 (0.6%)	0.95
Received transfusion	82 (2.1%)	124 (3.1%)	175 (2.9%)	0.007
Unplanned OR procedure	52 (1.3%)	50 (1.3%)	84 (1.4%)	0.81
Wound/postop complication	67 (1.7%)	92 (2.3%)	125 (2.1%)	0.12
Postpartum readmission	121 (3.0%)	163 (4.1%)	268 (4.5%)	0.001
Postpartum ICU admission	8 (0.2%)	3 (0.1%)	12 (0.2%)	0.26
Maternal death	1 (0.0%)	1 (0.0%)	1 (0.0%)	>0.99
*Neonatal morbidity composite*	833 (20.9%)	1015 (25.5%)	1404 (23.5%)	<0.001
APGAR at 5 min <4	107 (2.7%)	158 (4.0%)	223 (3.7%)	0.003
Neonatal death	30 (0.8%)	41 (1.0%)	47 (0.8%)	0.32
NICU admission	774 (19.4%)	940 (23.7%)	1285 (21.5%)	<0.001

Abbreviations: APGAR, Appearance, pulse, grimace, activity, respiration; GA, gestational age; ICU, intensive care unit; NICU, neonatal intensive care unit; OR, odds ratio; SVI, Social Vulnerability Index.

^a^ Defined as second trimester Hb < 10.5 g/dL/Hct < 32% or third trimester Hb < 11 g/dL/Hct < 33%.

**TABLE 3:** Outcomes with odds ratio and adjusted odds ratios among SVI groups.

	SVI (moderate) aOR (95% CI)	SVI (high) aOR (95% CI)^*^
Primary outcome		
Anemia^a^	1.11 (1.00–1.24)	1.18 (1.05–1.32)
Secondary outcomes		
*Labor outcomes*		
Mode of delivery^b^		
Vaginal	1.00 (0.90–1.11)	1.08 (0.98–1.20)
Cesarean	1.00 (0.91–1.11)	0.92 (0.84–1.02)
Delivery GA		
*Postpartum maternal morbidity composite* ^c^	1.46 (1.22–1.75)	1.47 (1.23–1.76)
Endometritis^d^	–	–
Received transfusion^e^	1.50 (1.13–1.99)	1.38 (1.06–1.80)
Unplanned OR procedure^f^	1.13 (0.75–1.70)	1.41 (0.95–2.09)
Wound/postop complication^g^	1.44 (1.03–2.01)	1.37 (0.97–1.92)
Postpartum readmission^h^	1.28 (1.00–1.66)	1.35 (1.05–1.75)
Postpartum ICU admission^i^	0.36 (0.10–1.35)	0.97 (0.40–2.38)
Maternal death^d^	–	–
*Neonatal morbidity composite* ^j^	1.38 (1.23–1.55)	1.32 (1.17–1.49)
APGAR at 5 min <4^k^	1.50 (1.16–1.95)	1.38 (1.06–1.80)
Neonatal death^l^	1.74 (1.07–2.83)	1.53 (0.91–2.57)
NICU admission^m^	1.36 (1.21–1.53)	1.29 (1.14–1.45)

*Note*: Reference = Low SVI. Data reported in *n* (%) and adjusted odds ratio (95% CI). Models did not converge for endometritis (*p* = 0.95) and maternal death (*p* > 0.99).

Abbreviations: aOR, adjusted odds ratio; APGAR, Appearance, pulse, grimace, activity, respiration; CI, confidence interval; GA, gestational age; GDM, gestational diabetes mellitus; ICU, intensive care unit; OR, odds ratio; NICU, neonatal intensive care unit; SVI, Social Vulnerability Index.

^a^ Adjusted for maternal age, race, marital status, payment source, and nulliparity.

^b^ Adjusted for maternal age, BMI, race, chronic hypertension, pregestational diabetes, and GDM.

^c^ Adjusted maternal age, BMI, race, chronic hypertension, pregestational diabetes, and drug use.

^d^ Everything was kicked out of the backwards selection model.

^e^ Adjusted for chronic hypertension and drug use.

^f^ Adjusted for maternal age, BMI, and race.

^g^ Adjusted for maternal age, BMI, race, pregestational diabetes, and drug use.

^h^ Adjusted for maternal age, BMI, race, payment source, and drug use.

^i^ Adjusted for pregestational diabetes.

^j^ Adjusted for BMI, race, marital status, payment source, chronic hypertension, pregestational diabetes, tobacco use, and aspirin use.

^k^ Adjusted for BMI, race, nulliparity, chronic hypertension, pregestational diabetes, GDM, drug use, and aspirin use.

^l^ Adjusted for race, chronic hypertension, tobacco use, and aspirin use.

^m^ Adjusted for BMI, race, marital status, payment source, chronic hypertension, pregestational diabetes, and aspirin use.

^*^Backward selection model with entry at *p* < 0.10.

Crude and adjusted analysis of secondary outcomes are demonstrated in Tables 2 and 3. When evaluating secondary outcomes, there were differences across SVI tertiles for mode of delivery, delivery GA, postpartum maternal morbidity composite (including transfusion and readmissions), and neonatal composite morbidity (Tables 2 and 3). For the individual postpartum maternal morbidity composite outcomes, the largest proportion of blood transfusion was in the moderate SVI group (3.1%, *p* = 0.007), while postpartum readmission had the largest proportion in the high SVI group (4.5%, *p* = 0.001).

In our secondary analysis, patients with anemia had a higher overall mean composite SVI scores than those without anemia (0.62 vs. 0.53, *p* < 0.001). This trend persisted across each of the four SVI themes (Table 4). When comparing each of the 15 individual components that compose the SVI score, the majority of individual elements were associated with anemia (Table 4).

**TABLE 4:** Mean Social Vulnerability Index scores by theme and individual components between groups.

Theme	SVI component	No anemia (*n* = 9871)	Anemia (*n* = 4080)	*p* value
Overall cohort, mean SVI score (95% CI)		**0.53 (0.52–0.53)**	**0.62 (0.61–0.62)**	**<0.001**
Theme 1: Socioeconomic status, mean (95% CI)		**0.48 (0.48–0.49)**	**0.58 (0.57–0.59)**	**<0.001**
	Persons below the poverty level	17.9 (9.9–28.3)	22.6 (13.6–32.1)	<0.001
	Persons unemployed	6.6 (3.8–12.0)	9.3 (4.8–13.5)	<0.001
	Per capita income, US dollars	23,439 (18,352–30,742)	20,644 (16,391–26,946)	<0.001
	Without a high school diploma	12.7 (6.9–19.4)	15.1 (9.1–20.4)	<0.001
Theme 2: Household composition, mean (95% CI)		**0.48 (0.48–0.49)**	**0.57 (0.56–0.58)**	**<0.001**
	Disabled	15.7 (11.3–20.9)	18.1 (12.9–23.0)	<0.001
	Persons >65 years	14.8 (11.2–18.0)	14.9 (11.3–18.0)	0.88
	Persons <18 years	23.1 (19.6–26.7)	23.1 (19.8–26.8)	<0.001
	Single‐parent households with children aged <18 years	9.6 (5.9–16.4)	11.8 (6.9–17.9)	<0.001
Theme 3: Minority status, language, mean (95% CI)		**0.63 (0.62–0.64)**	**0.66 (0.65–0.67)**	**<0.001**
	Persons of race or ethnicity other than non‐Hispanic White	46.7 (19.2–82.5)	71.1 (27.6–94.5)	<0.001
	Persons aged >5 years who speak English “less than well”	0.6 (0.0–1.7)	0.5 (0.0–1.5)	<0.001
Theme 4: Housing type and transportation, mean (95% CI)		**0.53 (0.53–0.54)**	**0.56 (0.55–0.57)**	**<0.001**
	Housing structure with >10 units	5.4 (0.6–15.9)	5.8 (0.9–13.7)	0.43
	Mobile homes	1.0 (0.0–8.9)	0.9 (0.0–7.1)	<0.001
	At household level, homes with more people than rooms	1.2 (0.1–2.5)	1.3 (0.3–2.6)	0.08
	Households with no vehicle available	6.0 (2.5–12.0)	8.1 (3.4–16.6)	<0.001
	Persons in institutionalized group quarters	0.3 (0.0–1.8)	0.3 (0.0–1.8)	0.44

Abbreviations: CI, confidence interval; SVI, Social Vulnerability Index.

Values are bold to deemonstrate the overall cohort and indivdiual theme *p*‐values.

When evaluating the strength of association of themes to anemia, the adjusted odds of anemia for each 1 unit change in SVI theme score are presented in Table 5. The adjusted odds ratios showed significant increased odds for every 1 unit change in SVI for theme 1 (socioeconomic status: aOR, 1.03; 95% CI, 1.02–1.05) and theme 2 (household composition and disability: aOR, 1.02; 95% CI, 1.01–1.04; Table 5).

**TABLE 5:** Unadjusted and adjusted odds ratios for maternal anemia conferred by each 1 unit change in the social vulnerability component.^a^

	Unadjusted OR (95%)	Adjusted OR (95%)^b^
Overall SVI	1.10 (1.08–1.11)	1.26 (1.09–1.45)
Theme 1: Socioeconomic status	1.11 (1.10–1.12)	1.03 (1.02–1.05)
Theme 2: Household composition and disability	1.09 (1.08–1.11)	1.02 (1.01–1.04)
Theme 3: Minority status and language	1.04 (1.02–1.05)	1.01 (0.99–1.02)
Theme 4: Housing type and transportation	1.04 (1.02–1.05)	1.00 (0.99–1.01)

Abbreviations: GA, gestational age; GDM, gestational diabetes mellitus; OR, odds ratio; SVI, Social Vulnerability Index.

^a^The continuous SVI themes have been rescaled to aid in interpretation by multiplying by 10. Therefore, a 1 unit change refers to 0.1 on the original SVI scale.

^b^Adjusted for maternal age, race, marital status, nulliparous, GDM, smoking during pregnancy, GA at delivery, preeclampsia, and abruption.

**FIGURE 2:**
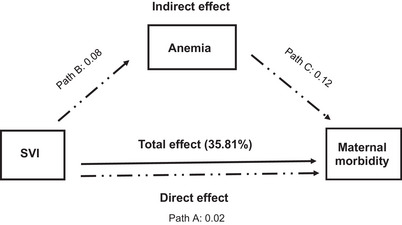
Mediation analysis for maternal morbidity. _SVI = independent variable. Anemia = mediator. Maternal morbidity = outcome. Maternal_ _composite—Path A: Logistic regression model, variance of logit:_ *_β_*_= 0.02,_ *_p_*_< 0.001. Path B:_ _Logistic regression model, variance of logit:_ *_β_*_= 0.08,_ *_p_*_< 0.001. Path C: Logistic regression_ _model, variance of logit:_ *_β_*_= 0.12,_ *_p_*_< 0.001. All paths: Logistic regression model, variance_ _of logit:_ *_β_*_= 0.12,_ *_p_*_< 0.001. Percentage mediated (95% confidence interval) = 35.81%__(22.91–60.92),_ *_p_*_value = <0.001. SVI, Social Vulnerability Index._

**FIGURE 3:**
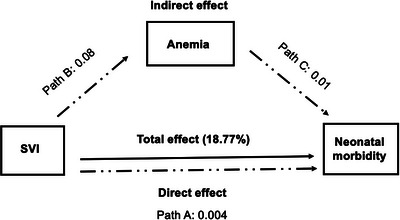
Mediation analysis for neonatal morbidity. _SVI = independent variable. Anemia = mediator. Neonatal morbidity = outcome. Neonatal_ _composite—Path A: Logistic regression model, variance of logit:_ *_β_*_= 0.004,_ *_p_*_= 0.001. Path B:_ _Logistic regression model, variance of logit:_ *_β_*_= 0.08,_ *_p_*_< 0.001. Path C: Logistic regression_ _model, variance of logit:_ *_β_*_= 0.01,_ *_p_*_< 0.001. All paths: Logistic regression model,_ *_β_*_= 0.01,_ *_p_*_< 0.001. Percentage mediated (95% confidence interval) = 18.77% (9.16–48.09),_ *_p_*_value = 0.009._ _SVI, Social Vulnerability Index._

Finally, to determine the contribution of anemia to poor perinatal outcomes associated with SVI, a mediation analysis was completed. This analysis found that 36% of the association between SVI and maternal morbidity was significantly mediated by anemia (*p* < 0.001; Figure 2), while only 19% of the association between SVI and neonatal morbidity was mediated by anemia (*p* = 0.009; Figure 3).

## 4 DISCUSSION

In this study, we found that patients living in census tracts with moderate and high levels of social vulnerability had higher odds of maternal anemia as well as select maternal and neonatal morbidity including postpartum readmission and blood transfusion. While all four themes (socioeconomic status, household characteristics, race and ethnicity minority status, house type transportation) that compose SVI differed between individuals with and without anemia, only Themes 1 (socioeconomic status) and Theme 2 (household composition) were significantly associated with increasing odds of anemia. Indicating that patients with higher rates of socioeconomic constraints or housing difficulties were identified to have higher odds of maternal anemia. These SVI themes help officials understand the specific needs of a community by categorizing vulnerability and allocating support by breaking down social factors. Further, while we found that the perinatal impact of SVI was mediated by anemia, the contribution was <50%, suggesting that there are other factors and variables outside of anemia contributing to adverse perinatal outcomes.

Pregnancy is a high‐risk time for the development of anemia due to a physiologic increase in plasma volume and increased nutritional needs. Variations in regional and global prevalence of anemia in pregnancy have previously been attributed to socioeconomic status and nutritional deficiencies [31, 32, 33]. Anemia in pregnancy and its adverse perinatal outcomes have been associated with low nutritional knowledge, food insecurity, low socioeconomic status and education, larger families, rural residence, and lack of health insurance [32, 33]. Our study supports these findings, but rather than looking at individual factors, we use a standardized measure of community‐level social vulnerability through the SVI. There is increasing evidence in pregnancy that supports the association of standardized measures of social vulnerability and adverse perinatal outcomes in preterm birth and severe maternal morbidity [13, 15, 34, 35]. Similar to preterm birth and maternal morbidity, one individual community component is unlikely to solely contribute to the development of anemia; therefore, an overarching assessment of an individual's community may perhaps better capture those at risk, incorporating all aspects of a community including those components unmeasurable on an individual level.

To our knowledge, there are no prior studies evaluating the effect of community‐level social vulnerability on the development of anemia in pregnancy. However, SVI has been associated with other adverse pregnancy outcomes, and literature on its use and other indices evaluating SDOH in pregnancy is increasing. A study by Givens et al. published in September 2021 evaluated the association between social vulnerability scores and preterm birth, discovering that those who live in areas of higher social vulnerability were more likely to deliver preterm [13]. More recently, Venkatesh et al. revealed that living in a community with higher social vulnerability was associated with a lower likelihood of achieving a hemoglobin A1c <6.0% during pregnancy with pregestational diabetes, and Pham et al. found an increased need for initiation glycemic agents in gestational diabetes in those with high SVI [19, 36]. There have also been associations between high SVI and increased odds of severe peripartum cardiomyopathy, as well as associations between high SVI and vaccine hesitancy in pregnancy [18, 37, 38]. Our analysis is unique compared to those previously mentioned as we also performed a mediation analysis that demonstrated that the association between SVI and maternal morbidity and SVI neonatal morbidity was significantly mediated by anemia—perhaps contributing to the association of the SVI and other adverse perinatal outcomes. Treatment of anemia (i.e., iron, transfusion) is a potential intervention to partially improve perinatal outcomes, and additional resources may need to be directed to areas that are socially vulnerable.

Clinicians may be aware that an individual patient's socioeconomic class or minority status places them at risk for anemia [4]. Our study further supports this notion that anemia in pregnancy may arise from many complex non‐biological social factors. Even with proper medical care, patients in communities with high level of social vulnerability remain at risk of anemia and adverse perinatal outcomes due to various influences and barriers inherent to their community [20, 22]. The SVI gives clinicians a tool to stratify pregnant individuals in regard to their risk of adverse perinatal outcomes.

Future prospective studies are needed to understand how routine assessment of the SVI may be integrated into patient care and the electronic medical record. If a patient with high SVI is identified, this could trigger additional screening, counseling, care coordination, treatment protocols, or referrals to help improve the identification and treatment of anemia in pregnancy. For example, screening patients by SVI can prompt incorporating iron studies into routine prenatal labs for those with high SVI, nutrition counselors for those in health care deserts, and determine barriers to treatment access such as transportation or a foreign language. Currently, there are no studies that have determined that earlier identification and treatment of anemia help improve anemia and associated perinatal outcomes [39].

However, our group recently demonstrated that pregnant patients with predelivery anemia were five times more likely to have a blood transfusion compared to those without anemia [40]. As a result, our institution has incorporated maternal serum ferritin levels at routine prenatal labs in the first and third trimesters. If either iron deficiency or iron deficiency anemia is detected, oral supplementation is recommended and if there is not an appropriate rise in ferritin after treatment or the patient is late in gestation, intravenous iron supplementation is recommended [41]. As ferritin level is used as a screening tool for anemia, SVI may further individualize the care needed to screen and treat patients with anemia and aid in the reduction of adverse perinatal outcomes. For example, for patients with high SVI, ferritin may be obtained more frequently, with prioritization of iron infusions with rapid resolution of anemia as opposed to oral iron medication.

While awaiting future study, our findings should encourage clinicians to use individualized approaches to the management of anemia in pregnancy for those patients with recognizable high levels of social vulnerability. For example, iron deficiency anemia has been traditionally treated with over‐the‐counter oral iron supplementation and relies heavily on medication compliance [6]. Earlier treatment with direct dispensing or intravenous iron infusions and nutrition education in conjunction with routine clinic appointments in those with high social vulnerability may eliminate the barrier of outpatient medication compliance, reduce anemia in pregnancy, and improve perinatal outcomes [42].

This study has multiple implications for research. As stated above, the use of SVI in a clinical setting deserves further investigation. It has yet to be determined whether increased screening or varied treatment modalities, such as increased and earlier use of IV iron infusions, for individuals with high SVI leads to improvement in anemia in pregnancy and associated perinatal outcomes. Our study found that all four themes that compose the SVI are associated with anemia, most notably low socioeconomic status (theme 1) and household composition (theme 2). Future inquiry into these individual themes of social vulnerability as the driving indicators of an SVI score to identify food deserts or malnourished patients may lead to more impactful targeted interventions in improving anemia in pregnancy. In addition, our study determined that while part of the perinatal impact of SVI is mediated through anemia, there must be other factors including SDOH not measured by SVI impacting this outcome. Research focusing on additional SDOH, including those at an individual rather than community level, is needed. Interestingly, in our study, patients living in areas of moderate social vulnerability had a greater odds of receiving a blood transfusion, experiencing postpartum wound complications, and increased overall neonatal morbidity compared to those living in areas with high social vulnerability. It can be postulated that an increasing effect was not seen across tertiles as those with highest vulnerability may have had more comorbidities leading to more in‐depth medical care. More comprehensive analysis would be required to confirm or deny this hypothesis.

Our findings should be interpreted within the context of the study limitations. We acknowledge that our patient population was limited to the data collected at a single tertiary care center, which may limit the generalizability; however, as a referral center, it represents a large geographically diverse area increasing generalizability. As the SVI ranking score was determined by home address at the time of delivery for patients, we were unable to account for housing instability and patients moving across SVI classes. Of note, our study excluded patients who only reported a PO Box address as this group could not be assigned an SVI, but this excluded group could be inherently different, resulting in a higher or lower SVI than the present population due to housing instability. Also, anemia was defined based on second and third trimester values at delivery and not in the first trimester. Our analysis was limited to deliveries ≥ 23 weeks, but we should acknowledge that we were unable to examine the relationship between SVI and early anemia in pregnancy as well as the contribution of early anemia to adverse perinatal outcomes. Finally, a lack of accessible data precluded an assessment of prenatal care adequacy and the specific considerations of treatment protocols for iron deficiency anemia, including the route of iron administration (oral or intravenous).

Our study has several strengths. First, our study had a large sample size across the SVI groups that spanned census tracts. Second, we used a previously validated and published database with individual review of certain outcomes and validated abstraction for other outcomes [24, 25]. Third, our study was conducted at a referral tertiary care center representing a large, geographically diverse region [24].

## 5 CONCLUSIONS

In summary, our study demonstrated that patients living in moderate and high SVI geographic regions are more likely to have anemia and other adverse perinatal outcomes. This suggests that SVI could potentially serve as a clinical tool for the creation of targeted interventions, such as the inclusion of iron studies, to reduce the risk of adverse perinatal outcomes in disparate communities. The perinatal impact of SVI is mediated through anemia, but other factors including potentially individual level SDOH or clinical factors likely also contribute to adverse pregnancy outcomes. Future studies exploring the clinical usability of the SVI and the development of targeted interventions for those with high social vulnerability are needed to further identify and treat patients at high risk of maternal anemia and adverse maternal and neonatal outcomes.

## CONFLICT OF INTEREST STATEMENT

A.N.B. was supported by NICHD K23HD103875 during the study. The other authors declare no conflicts of interest.

## FUNDING INFORMATION

The authors received no specific funding for this work.

## ETHICS STATEMENT

This study was approved by the Institutional Review Board (IRB) of the University of Alabama at Birmingham under IRB protocol number 300002492.

## Data Availability

The datasets used during the current study are available from the corresponding author on reasonable request.
